# ﻿Traces of past reintroduction in genetic diversity: The case of the Balkan chamois (Mammalia, Artiodactyla)

**DOI:** 10.3897/zookeys.1116.84577

**Published:** 2022-08-04

**Authors:** Andrea Rezić, Toni Safner, Laura Iacolina, Elena Bužan, Nikica Šprem

**Affiliations:** 1 University of Zagreb, Faculty of Agriculture, Department of Fisheries, Apiculture, Wildlife Management and Special Zoology, Svetošimunska c. 25, 10000, Zagreb, Croatia; 2 University of Zagreb, Faculty of Agriculture, Department of Plant Breeding, Genetics and Biometrics, Svetošimunska c. 25, 10000, Zagreb, Croatia; 3 Centre of Excellence for Biodiversity and Molecular Plant Breeding (CoE CroP-BioDiv), Svetošimunska c. 25, 10000, Zagreb, Croatia; 4 University of Primorska, Faculty of Mathematics, Natural Sciences and Information Technologies, Department of Biodiversity, Glagoljaška 8, 6000, Koper, Slovenia; 5 Aalborg University, Department of Chemistry and Bioscience, Section of Biology and Environmental Science, Fredrik Bajers Vej 7H, 9220 Aalborg East, Denmark; 6 Faculty of Environmental Protection, Trg mladosti 2, 3320, Velenje, Slovenia

**Keywords:** Biokovo, genetic structure, microsatellite, Prenj, translocation

## Abstract

The translocation of wild animal species became a common practice worldwide to re-establish local populations threatened with extinction. Archaeological data confirm that chamois once lived in the Biokovo Mountain but, prior to their reintroduction in the 1960s, there was no written evidence of their recent existence in the area. The population was reintroduced in the period 1964–1969, when 48 individuals of Balkan chamois from the neighbouring mountains in Bosnia and Herzegovina were released. The main objective of this study was to determine the accuracy of the existing historical data on the origin of the Balkan chamois population from the Biokovo Mountain and to assess the genetic diversity and population structure of the source and translocated populations 56 years after reintroduction. Sixteen microsatellite loci were used to analyse the genetic structure of three source chamois populations from Prenj, Čvrsnica and Čabulja Mountains and from Biokovo Mountain. Both STRUCTURE and GENELAND analyses showed a clear separation of the reintroduced population on Biokovo from Prenj’s chamois and considerable genetic similarity between the Biokovo population and the Čvrsnica-Čabulja population. This suggests that the current genetic composition of the Biokovo population does not derive exclusively from Prenj, as suggested by the available literature and personal interviews, but also from Čvrsnica and Čabulja. GENELAND analysis recognised the Balkan chamois from Prenj as a separate cluster, distinct from the populations of Čvrsnica and Čabulja. Our results thus highlight the need to implement genetic monitoring of both reintroduced and source populations of endangered Balkan chamois to inform sustainable management and conservation strategies in order to maximise the chances of population persistence.

## ﻿Introduction

The reintroduction and translocation of wild species for various purposes became a common practice worldwide and was used as a conservation tool for rescuing and re-establishing extirpated populations (Cullingham and Moehrenschlager 2013). Northern chamois (*Rupicaprarupicapra* L.) is one of the examples of successfully translocated species (Apollonio et al. 2014) in many areas of Europe (Crestanello et al. 2009; Martínková et al. 2012; Šprem and Buzan 2016), but also on other continents such as South America (Corlatti et al. 2011) and New Zealand (Christie 1964). Past translocations of chamois left a genetic signature in recent populations which can be now used for reconstructing undocumented events (Crestanello et al. 2009).

Today’s populations of the Northern chamois in northern Dinaric Mountains in Croatia are descendants of successfully translocated individuals captured on mountain areas in Bosnia and Herzegovina (*Rupicaprarupicaprabalcanica*) and Slovenia (*Rupicaprarupicaprarupicapra*) (Apollonio et al. 2014; Šprem and Buzan 2016). Since different subspecies were involved in past reintroduction efforts, a contact zone was formed on the northern Velebit Mountains where these subspecies hybridise (Šprem and Buzan 2016).

The Balkan chamois (*Rupicaprarupicaprabalcanica*) is one of the seven recognised subspecies of the Northern chamois. It is found both in the mountainous regions of Croatia and in the mountain ranges of the eight other countries of the Balkan Peninsula, from north to south: Bosnia and Herzegovina, Serbia, Montenegro, Kosovo, North Macedonia, Albania, Bulgaria, and Greece. The lack of continuity of these habitats and overhunting in the post-Neolithic period have severely fragmented the subspecies’ present distribution (Corlatti et al. 2022). In addition to low colonisation rates and reduced gene flow between isolated populations, which may lead to genetic differentiation due to the inbreeding effect and loss of allelic variants, the Balkan chamois is threatened by poaching (Papaioannou and Kati 2007), habitat change (Kavčić et al. 2019), unsustainable hunting (Šprem and Buzan 2016) and by the introduction of Alpine chamois subspecies (Iacolina et al. 2019). As a conservation measure, the Balkan chamois is listed in Annexes II and IV of the European Union Habitats Directive 92/43/EEC (OJ L 206, 22.7.1992) and in Appendix III of the Bern Convention (OJ L 38, 10.2.1982). The Balkan chamois is one of the most poorly studied subspecies of Northern chamois and knowledge on the genetic diversity and structure of the Balkan chamois population is limited and restricted to regional-local studies (Markov et al. 2016; Šprem and Buzan 2016; Papaioannou et al. 2019; Rezić et al. 2022).

The genetic structure of the Balkan chamois population on the Biokovo has been studied only by Šprem and Buzan (2016), and few other ecological studies have included this population in a population density estimation (Kavčić et al. 2021a) and rutting behaviour (Kavčić et al. 2021b). The study of phylogenetic relationships in Šprem and Buzan (2016) revealed the existence of endemic Balkan haplotypes in the Prenj and Biokovo Mountains and a genetic richness of the historically viable Prenj population comparable to Alpine chamois studied in Buzan et al. (2013) from the south-eastern Alps. The paleontological findings in the Baba cave, which are more than ten thousand years old, confirm that chamois once lived in Biokovo (Šabić 2011) but, before the reintroduction in the 1960s, there was no written evidence of the recent existence of chamois in this area. According to historical records, the chamois on Biokovo Mt. are descendants of individuals translocated from the “Prenj” hunting district in Bosnia and Herzegovina established in 1961 (Rapaić and Kunovac 2020) which included both Prenj and Čvrsnica massifs. This hunting district had, at that time, stable and numerous chamois populations and was used for many reintroduction programmes in the Balkans (Rapaić and Kunovac 2020). The reintroduction of chamois on Biokovo Mt. was the result of the planned introduction on this area by the Union of Hunting Association, the municipality of Makarska and the Union of Hunting Association of Imotski, mainly with the aim of increasing the population size for hunting purposes (Šabić 2011). Prior to the reintroduction from the “Prenj” hunting district, assessment of the suitability of the Biokovo habitat was made and, after a positive evaluation, a first release of 7 individuals (3 males and 4 females) took place on 1 November 1964 (Šabić 2014). A total of 48 chamois was successfully reintroduced in the period between 1 November 1964 and 23 October 1969 (Šabić 2014). The success of the reintroduction of the Balkan chamois in the Biokovo Mt. is reflected by the latest population size estimate (Kavčić et al. 2021a), according to which this area is now inhabited by at least 600 individuals.

The main objective of this study was to determine the accuracy of historical data on the origin of chamois in Biokovo, and to assess and document the genetic status of both the source and translocated populations, 56 years after reintroduction, by using microsatellite markers.

## ﻿Materials and methods

### ﻿Ethical statement

All samples used in this study were from hunted (regular hunting activities approved by the competent Ministry of Agriculture of the Republic of Croatia within the annual game management plans) and from remains of naturally dead animals (samples from Bosnia and Herzegovina).

### ﻿Population sampling

**Figure 1.**
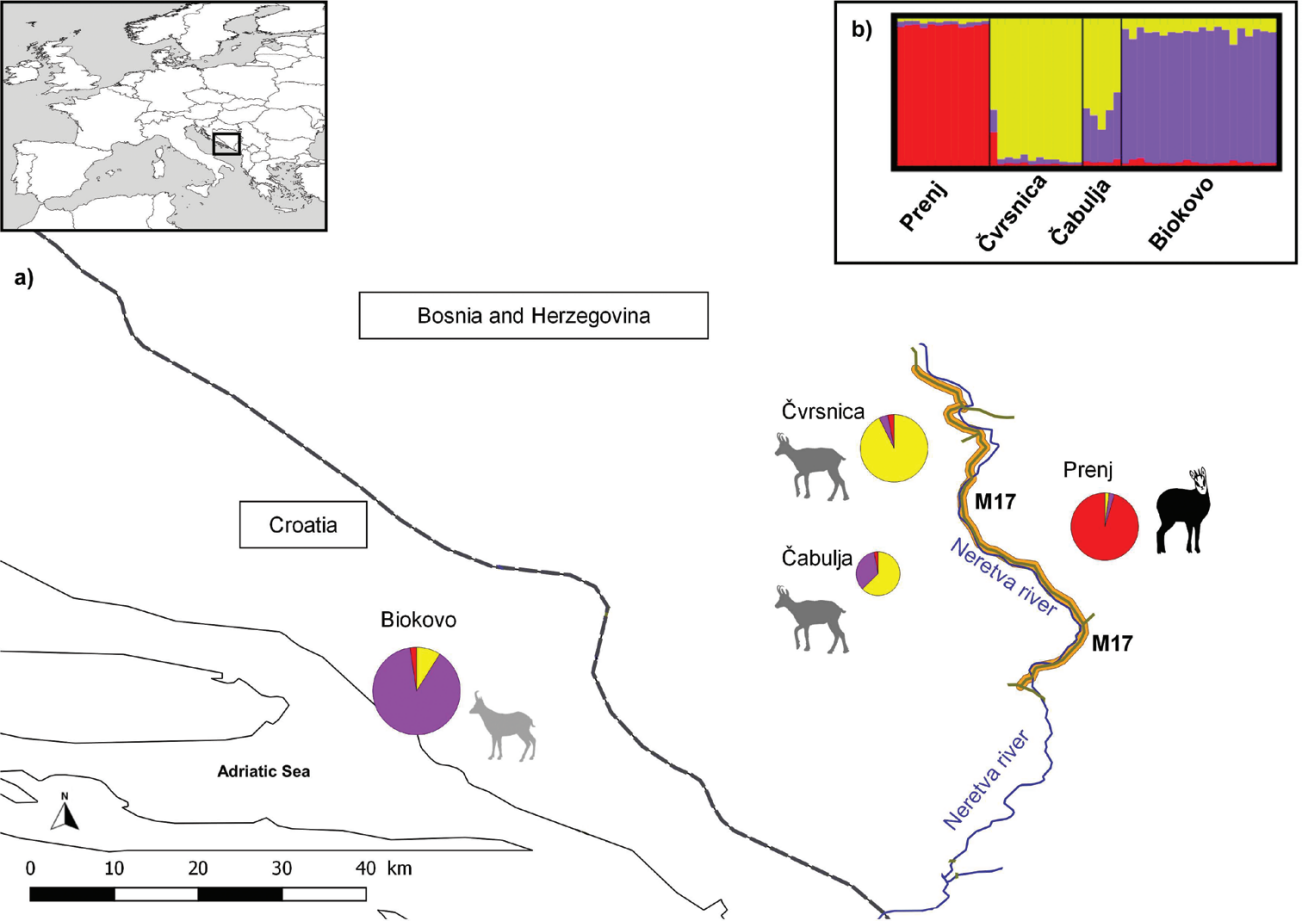
Results of the analysis of sixteen microsatellite loci in four Balkan chamois populations **a** geographical representation of results from STRUCTURE and GENELAND software. The pie charts show the results from STRUCTURE for *K* = 3. The different colours of the pie charts represent the proportions of each ancestral genotype per individual *q* (in %) in each of the four predefined Balkan chamois populations. The size of the pie charts indicates the number of samples collected at each location. The different shapes and colours of the chamois silhouettes represent the results of the spatial analysis under uncorrelated frequency model performed in GENELAND. The three spatial clusters are shown, while the assignment to the fourth ghost cluster was not shown because no individuals were assigned to it (see text for details). The dashed line indicates the national border, while the state road M17 in Bosnia and Herzegovina is marked with an orange line. The green lines represent connections with other main roads. The course of the river Neretva is marked by a blue line **b** genetic structure of the 49 Balkan chamois individuals analysed, shown as a bar plot from STRUCTURE at *K* = 3. Each vertical bar represents an individual, and the percentage of each colour corresponds to the percentage of the respective ancestral genotype. The studied populations are separated by a black line.

We collected 20 samples from Biokovo and 29 samples from three areas which serve as source populations for reintroduction and possible recent recolonisation (Prenj, Čvrsnica, and Čabulja Mountains). Details of sampling locations are given in Fig. 1 and Table 1. The samples were collected between 2017 and 2020. After collection, the samples were preserved in 96% ethanol, delivered and stored at -80 °C in the Laboratory of molecular ecology, University of Primorska.

**Table 1.** Genetic diversity of four Balkan chamois populations assessed using sixteen microsatellite loci.

Population locality/ country	*N*	*H_O_* (SD)	*H_E_* (SD)	HWE	*F_IS_* (*IC* 95%)	*A*	*AR*	*N_pr_*	*N_e_* (*IC* 95%)
Prenj 43°32'03"N, 17°54'12"E/BIH	12	0.636 (0.274)	0.637 (0.150)	0.013*	0.046 (-0.147–0.116)	4.500	2.517	11	10.500 (6.500–18.600)
Čvrsnica 43°38'18"N, 17°38'30"E/BIH	12	0.552 (0.287)	0.536 (0.206)	0.011^NS^	0.014 (-0.131–0.038)	3.937	2.223	3	7.700 (4.100–13.600)
Čabulja 43°29'11"N, 17°37'20"E/BIH	5	0.575 (0.251)	0.535 (0.187)	0.913^NS^	0.059 (-0.415–0.089)	3.375	2.376	2	–
Biokovo 43°19'47"N, 17°63'05"E/HRV	20	0.584 (0.132)	0.597 (0.138)	0.389^NS^	0.048 (-0.050–0.084)	4.625	2.356	6	57.200 (28.600–371.800)

BIH – Bosnia and Herzegovina, HRV – Croatia; *N* – number of samples; *H_O_* – observed heterozygosity; *H_E_* – expected heterozygosity; SD – standard deviation; *F_IS_* – inbreeding coefficient; *IC* 95% – 95% Confidence Interval; HWE – Hardy-Weinberg equilibrium (after Bonferroni adjustment *p* values: ^NS^ – non-significant value; * – significant at *p* < 0.05); *A* – average number of alleles; *AR* – allelic richness; *N_pr_* – number of private alleles; *N_e_* – effective population size and its confidence interval estimated with the chi-square (i.e., parametric).

### ﻿DNA extraction and microsatellite amplification

We extracted DNA from tissue samples (*N* = 49) using the commercial peqGOLD Tissue DNA Mini Kit (PEQLAB Biotechnologie GmbH) following the manufacturer´s protocol in a final volume of 150 µL. DNA concentrations were measured with Qubit dsDNA BR Assay Kit (Invitrogen BR Assay Kit, Carlsbad, CA, USA) on a 3.0 Qubit Fluorimeter (Life Technologies, Carlsbad, CA, USA). Sixteen microsatellites were amplified using PCR multiplex sets previously investigated in studies with chamois (Zemanová et al. 2011; Buzan et al. 2013; Šprem and Buzan 2016; see Suppl. material 1: Table S1). The PCR protocol described in Rezić et al. (2022) was used for amplification of microsatellite regions. Genotyping errors were assessed by re-genotyping of ten randomly chosen individuals from the final data set and comparing these genotypes to the initial ones. Fragment analysis was performed on an ABI 3130 Genetic Analyzer (Applied Biosystems) using the GeneScan LIZ500 (-250) Size Standard (Applied Biosystems). Microsatellite genotypes were analysed using Gene Mapper v. 4.0 software (Applied Biosystems).

### ﻿Microsatellite data analysis

We used the Expectation-Maximization (EM) algorithm implemented in FREENA (Chapuis and Estoup 2007) to estimate null allele frequencies for each microsatellite locus, as they can cause significant heterozygote deficit and population deviation from Hardy-Weinberg equilibrium (HWE). Values of null allele frequency greater than *r* ≥ 0.20 were reported (see Suppl. material 1: Table S2). FREENA software was also used to calculate global *F_ST_* values and *F_ST_* values for each pair of analysed populations, both with and without the use of the excluding null alleles (ENA) correction method, as described in Chapuis and Estoup (2007). The Wilcoxon Two Sample test was used to compare the corrected *F_ST_* values with the original *F_ST_* values and to test the significance of null alleles in the analyses. The Wilcoxon Two Sample test was performed in R ver. 4.0.5 package stats (R Core Team 2020).

We considered each sampling location as a separate population due to limited dispersal of subspecies between mountain ranges (see Table 1). The exact probability test for each locus and population was used to test the deviation of the observed genotype frequency from HWE using the Markov chain method with 10,000 dememorisation steps, 500 batches and 10,000 subsequent iterations in GENEPOP ver. 4.7.2 (Rousset 2008). The same test, based on a Markov chain method implemented in Genepop, was used to analyse pairwise linkage disequilibrium (LD) between all pairs of loci in all populations. A sequential Bonferroni procedure (Holm 1979) was applied to correct for the effect of multiple comparison tests by using the adjust *p*-values function implemented in R ver. 4.0.5 package stats.

GENETIX ver. 4.05.2 (Belkhir et al. 1996–2004) was used to calculate the mean number of alleles, observed (*H_O_*) and expected (*H_E_*; Nei 1978) heterozygosity for each locus in all populations, and the inbreeding coefficient (*F_IS_*) and its confidence intervals. The number of private alleles was estimated using the GENALEX ver. 6.502 (Peakall and Smouse 2012). We estimated allele richness in each population using the rarefaction procedure implemented in FSTAT ver. 2.9.3.2 (Goudet 2001). The same software was used to analyse the level of genetic differentiation between sampling populations (pairwise F_ST_) and calculate their respective *p*-values using 1000 permutations.

The Bayesian clustering program STRUCTURE ver. 2.3.4. (Pritchard et al. 2000) was used to estimate the most likely number of ancestral genotypes (*K*) within the entire sample, and to estimate the proportions of each ancestral genotype in Balkan chamois individuals. We ran the analysis allowing for admixture and correlated allele frequency with ten independent runs for each *K* between 1 and 7 with a burn-in 500,000 steps followed by 10^5^ Markov chain Monte Carlo (MCMC) iterations. The results of the repeated runs for each value of *K* were combined with the Greedy algorithm in CLUMPP v. 1.1.2 (Jakobsson and Rosenberg 2007), and the summary outputs were visualised with DISTRUCT v. 1.1 (Rosenberg 2004). To estimate the most likely *K*, we applied the ad hoc summary statistic ∆*K* developed by Evanno et al. (2005). STRUCTURE HARVESTER (Earl and vonHoldt 2012) was used to compare the average estimates of the likelihood of the data, *ln*[Pr(X|K)] for each value of *K.* The same software was used to generate graphs for the mean log posterior probability of the data (mean ± SD).

The modal proportions of ancestral genotypes for each individual in each sampled area from the run with the highest log-likelihoods was plotted on a map using QGIS ver. 2.18.21 (QGIS Development Team 2018). We estimated the effective population size (*N_e_*) using the linkage disequilibrium-based method (Hill 1981; Waples 2006; Waples and Do 2010) implemented in NeESTIMATOR V2 (Do et al. 2014). Rare alleles below an allele frequency of 0.02 were excluded (as recommended by Waples and Do 2010). The effective population size for Čabulja Mt. was not calculated due to the small sample size.

The robustness of the results of STRUCTURE was estimated by analysing the same data with the spatial Bayesian clustering model implemented in GENELAND software (Guillot et al. 2005a). Although there are several Bayesian clustering methods that perform spatial analysis of genetic data, GENELAND was chosen because it provides the most accurate estimates of true genetic structure (Safner et al. 2011). We followed the recommendations of Guillot et al. (2005a) to set up the analysis. The algorithm was run in two steps. In the first step, the algorithm was run ten times to infer *K* under the uncorrelated frequency model, with the parameter indicating the degree of uncertainty of the spatial coordinates set to 10. The MCMC iterations were set to 10^6^ with a thinning of 100. The number of populations was set from *K* = 1 to *K* = 5. The maximum number of nuclei in the Poisson-Voronoi tessellation was set to 300. After determining the number of population clusters in the first step, we ran the algorithm setting *K* to this number and leaving the other parameters as in the first step.

## ﻿Results and discussion

The sixteen microsatellite loci yielded a total of 95 alleles, which varied between 2 (for locus ETH10 and SR-CRSP-6) and 10 (for locus BM1258) with an average value of 5.937 alleles per locus (see Suppl. material 1: Table S1).

The values of null allele frequencies were low for most analysed loci, except for loci ETH10, SY434, TGLA53, and SR-CRSP-6, whose frequencies were estimated to be *r* ≥ 0.20 (see Suppl. material 1: Table S2). The presence of null alleles was found in all three populations from Bosnia and Herzegovina. The various factors caused by natural population mechanisms, such as disassortative mating, bottleneck, fluctuations in population size, can cause heterozygote deficit that can be interpreted as false positive presence of null alleles (Dąbrowski et al. 2014), which led to the decision to retain all analysed loci.

The Prenj population deviated from Hardy-Weinberg equilibrium (HWE) but the deviation was significant at the 0.05 level only for locus SY434 after applying sequential Bonferroni adjustment (Table 1). This may be a consequence of the recent severe bottleneck in this population, which was previously stable. Gafić and Džeko (2009) noted that the population of Balkan chamois in the Prenj hunting district, which included both the Prenj and Čvrsnica massifs, was approximately 4,000 individuals in 1966, but due to the civil war in the 1990s, the population was greatly reduced by illegal hunting, with up to 95% of the population lost. Since no deviation from HWE was observed at this locus in other populations, we retained it in all subsequent analyses. After applying the sequential Bonferroni correction to the linkage disequilibrium results, no significant value was observed.

The Prenj population had the highest values of observed (0.636) and expected (0.637) heterozygosity, and allelic richness (2.517). A similar pattern was recorded in the study of Šprem and Buzan (2016) where the Prenj population had the highest values of allelic richness, observed, and expected heterozygosity and significantly deviated from HWE. In the Šprem and Buzan (2016) study, the Biokovo population had the lowest allelic richness. The observed number of alleles (*A*) varied from 3.375 in the Čabulja population to 4.625 in the Biokovo population. All populations had private alleles (*N_pr_*) and the highest number of private alleles (11) was observed in the Prenj population from Bosnia and Herzegovina (Table 1).

Effective population size was estimated for three sampled sites, excluding the Čabulja population due to small sample size (Table 1). Čvrsnica had the lowest results for *N_e_*, although very similar to those estimated for Prenj. The higher estimates for the Biokovo population should be taken with caution, considering our results suggest the presence of multiple funding sources. Additionally, Do et al. (2014) showed that microsatellite loci could lead to a slight upward bias for the linkage disequilibrium method when the critical value is set to *p* = 0.02.

The lowest *F_ST_* value was found between Čvrsnica and Čabulja (*F_ST_* = 0.024), while the highest and significant *F_ST_* value (0.084) was observed between two neighbouring populations from Bosnia and Herzegovina (Prenj and Čvrsnica; Table 2). The global *F_ST_* values were 0.067 (*CI* = 0.031–0.108) without using the correction method and 0.071 (*CI* = 0.038–0.112) with the ENA correction method for null alleles. The Wilcoxon Two Sample test showed no significant differences between the corrected and original *F_ST_* values (*p* = 0.734), indicating that the presence of putative null alleles did not affect the analysis (see Suppl. material 1: Fig. S1).

**Table 2.** Pairwise F_ST_ values between four studied populations of Balkan chamois.

Populations	Čvrsnica	Čabulja	Biokovo
Prenj	0.084*	0.047^NS^	0.074*
Čvrsnica		0.024^NS^	0.072*
Čabulja			0.027^NS^

*p* values: ^NS^ – non – significant value; * - significant at *p* < 0.05.

The algorithm developed by Evanno et al. (2005) identified *K* = 3 as the optimal number of ancestral genotypes detected by STRUCTURE analysis for four analysed populations and detected another peak at *K* = 5 suggesting a possible further genetic structure subdivision within the populations (see Suppl. material 1: Fig. S2). According to STRUCTURE results, populations from Prenj, Čvrsnica, and Biokovo had high proportions of genomes from a single ancestral genotype (*q* ≥ 0.88 in all three populations), which for Prenj differ from Čvrsnica and Biokovo, while in the population from Čabulja the highest proportion of same ancestral genotype as in Čvrsnica was lower (*q* = 0.63; Fig. 1a). The difference in genetic composition between Balkan chamois from Prenj and other populations is likely due to a barrier to gene flow between the studied populations, but also probably a consequence of recent bottleneck effect, due to extirpation of chamois during the Balkan civil war (Frković 2008) and population local adaptation. Čvrsnica, Čabulja, and Prenj Mts. are restricted to habitat patches, particularly by the steep river canyon of Neretva, which might lead to their fine-scale fragmentation. The Neretva River, one of the largest rivers in the eastern part of the Adriatic Basin, separates the Prenj Mt. from the Čvrsnica and Čabulja Mts. and forms the official border between the “Čvrsnica” and “Prenj” game reserves (Jurić 1998). Landscape features of the Neretva River valley, as well as the adjacent road M17, built as a part of the European Route E73, can act as effective barriers for the species natural dispersal, and hinder gene exchange between the studied populations (Soglia et al. 2010; Buzan et al. 2013). Additionally, the population decline in the last Balkan civil war could have contributed by isolating small groups of chamois to restricted habitat patches, although geographically very close to each other. Individual proportions of ancestral genotypes assigned by STRUCTURE support the higher levels of admixture in Čabulja when compared to the other populations (Fig. 1b). According to the *q* values, only one individual from Čabulja had a *q* value above the threshold of 0.75 sharing similar genotype as individuals from the Čvrsnica population, while all others had admixed genotype (*q* < 0.70). One individual from Čvrsnica stood out from the others with a *q* = 0.23 proportion of ancestral genotype that was present mostly in Prenj population. As previously mentioned, natural migration between individuals from Prenj and Čvrsnica is currently unlikely, due to the presence of barriers, however this might not have been the case before the construction of the road infrastructure. STRUCTURE indicated that the reintroduced Balkan chamois population in the Biokovo Mt. is genetically more similar to the population from Čabulja, suggesting that the reintroduced individuals in the Biokovo Mt. may originate from this area, as well as from Čvrsnica, whereas animals translocated from Prenj did not leave detectable genetic signatures. According to Jurić (1998), 992 individuals of Balkan chamois were counted in the Čvrsnica hunting ground which also includes the territories of the neighbouring Čabulja Mt. It is possible that populations Čvrsnica and Čabulja were connected in the past and formed a single population, as can be inferred from the results of STRUCTURE. Due to the civil war in the Balkans in the 1990s and continued illegal hunting and poaching, this single population has dwindled in numbers and become fragmented and isolated in the high mountain habitats (statements of local people). The divergence between Prenj and Biokovo from Čvrsnica and Čabulja populations may be due to the historical founder effect and more recent genetic drift due to isolation, and local adaptation. Despite decades of long unsustainable hunting, predation, poaching and natural events (Šprem and Buzan 2016) the inbreeding levels in the analysed populations are still moderate. The results of genetic composition of Biokovo population can influence population viability through time and it is very important to monitor genetic parameters of the reintroduced population to prevent a loss of genetic diversity due to inbreeding and genetic drift (DeMay et al. 2017).

To improve the previous analyses, the spatial context of individuals was taken into consideration and tested with GENELAND. This analysis revealed a similar pattern of clustering of individuals as STRUCTURE, but suggested an additional fourth spatial cluster along the MCMC chain (see Suppl. material 1: Fig. S3). GENELAND detected two spatial clusters among the three analysed populations in Bosnia and Herzegovina, while one spatial cluster corresponded to the population in Biokovo (Fig. 1). The distinction between Prenj and Biokovo was also detected by Šprem and Buzan (2016), who used the BAPs algorithm for spatial clustering of groups and showed the separation of the Balkan chamois population of the Prenj Mt. from the Biokovo Mt. population. The fourth cluster revealed by GENELAND spatial model was a so-called “ghost cluster” (Frantz et al. 2009) since no individual was assigned to it. Ghost clusters are not uncommon but are still a poorly understood phenomenon that can be caused by a heterogeneous distribution of samples (Aziz et al. 2018). It is possible that all the clusters identified by GENELAND represent a true genetic subdivision, but the degree of differentiation between them was too low for the clustering to be consistent (Frantz et al. 2009). Another possibility is that the GENELAND model might overestimate the number of genetic clusters when analysing populations which are affected by isolation by distance (Frantz et al. 2009).

Future studies will need to incorporate non-invasive genetic sampling, telemetry and behavioural patterns to confirm possible migration and gene flow between these populations. In the available literature there is no indication of the exact location where the animals released on Biokovo were caught. It is only known that the individuals came from the Prenj hunting district, which included two game reserves called “Čvrsnica” and “Prenj” (established in 1893 by the Austro-Hungarian Empire) and which were declared protected areas (Rapaić and Kunovac 2020). Jurić (1998) stated in his master’s thesis that the translocation of Balkan chamois from the game reserve “Čvrsnica” began in 1962 and lasted until 1970. During this period, a total of 101 Balkan chamois was translocated to different areas in the Balkans, but the author did not write any additional information about the location where the animals were caught or the places where they were translocated. It is not yet known whether these data exist. Therefore, it is very important to establish standards for documenting and monitoring species translocation projects.

## ﻿Conclusions

Non-invasive monitoring of genetic parameters of both reintroduced and source populations of endangered Balkan chamois, together with demographic monitoring, is crucial for sustainable management practices and improving conservation strategies to maximise the chances of population persistence. Our genetic diversity results show that the Balkan chamois population from Biokovo can serve as a potential source for future translocations, especially to the source habitats, Čvrsnica and Čabulja, that are currently threatened by loss of genetic diversity due to unsustainable hunting and poaching, leading to inbreeding and genetic drift.

## Supplementary materials

Supplementary material 1 Tables and figures Data type Population genetics. Explanation note **Table S1**. Information about microsatellite primer sequences used for the analysis of Balkan chamois population genetics. **Table S2**. Locus/population matrix containing null alleles identified by the FreeNA software. Null allele frequencies were estimated using the EM algorithm. Frequencies with values higher than r ≥ 0.20 were indicated. **Figure S1**. The comparison of two groups of FST values (original pairwise FST values and ENA-corrected pairwise FST values) using the Wilcoxon Two Sample test. **Figure S2**. Evanno method (Evanno et al. 2005) for selecting the representative number of clusters (K). **Figure S3**. Number of clusters along the MCMC chain for spatial analysis performed with the GENELAND software. https://binary.pensoft.net/file/723161This dataset is made available under the Open Database License (http://opendatacommons.org/licenses/odbl/1.0/). The Open Database License (ODbL) is a license agreement intended to allow users to freely share, modify, and use this Dataset while maintaining this same freedom for others, provided that the original source and author(s) are credited.Andrea Rezić, Toni Safner, Laura Iacolina, Elena Bužan, Nikica Šprem
