# Supplementary material for: ﻿Traces of past reintroduction in genetic diversity: The case of the Balkan chamois (Mammalia, Artiodactyla)
Source: Zookeys. 2022 Aug 4;1116:57–70. doi: 10.3897/zookeys.1116.84577 (PMC9848663; doi:10.3897/zookeys.1116.84577)
Supplement: Supplementary material 1 — Tables and figures [file zookeys-1116-057_article-84577__-s001.pdf]

Table S.1. Information about microsatellite primer sequences used for the analysis of Balkan chamois population genetics.

Multiplex sets	Locus	Size range (bp)	Accession no.	C (µL)	*Na*	References
SET 1	OarFCB20	80-104	L20004	0.3	5	Buchanan et al. 1994,
	OarFCB304	130-146	L01535	0.3	8	Buchanan & Crawford 1993
	SR-CRSP-5	154-174	L22197	0.2	9	Arevalo et al. 1994
	SY84	170-180	AY725829	0.3	5	An et al. 2005
	CSSM66	193-255	AF232764	0.3	7	Barendse et al. 1994
	ETH10	205-213	Z22739	0.3	2	Toldo et al. 1993
SET 2	SY434	83-101	AY725834	0.3	7	An et al. 2005
	SR-CRSP-11	112-132	-	0.2	7	Kogi et al. 1995
	TGLA53	132-156	GQ368903	0.3	3	Barendse et al. 1994
	BOBT24	148-176	-	0.3	8	Buitkamp et al. 1996
SET 3A	BM1258	101-129	G18385	0.2	10	Bishop et al. 1994
	SR-CRSP-9	124-132	L22000	0.3	7	Bhebhe et al. 1994
	ILSTS030	152-180	L37212	0.3	6	Kemp et al. 1995
	ETH225	138-150	Z14043	0.3	6	Steffen et al. 1993
SET 3B	SR-CRSP-6	140-150	L22198	0.3	2	Bhebhe et al. 1994
	NRAMP1	196-218	AF005380	0.4	3	Matthews & Crawford 1998
				**TOTAL**	**95**	
				**AVERAGE**	**5.94**	

C – primer concentration (in microliter), stock solution was 100 µM; Na - total number of alleles for each locus

Table S.2. Locus/population matrix containing null alleles identified by the FreeNA software. Null allele frequencies were estimated using the EM algorithm. Frequencies with values higher than $r \geq 0.20$ were indicated.

	**Populations**				
**Locus**	**PRENJ**	**ČVRSNICA**	**ČABULJA**	**BIOKOVO**	**Total**
OarFCB20	-	-	-	-	0
OarFCB304	-	-	-	-	0
SR-CRSP-5	-	-	-	-	0
SY84	-	-	-	-	0
CSSM66	-	-	-	-	0
ETH10	0.209	-	-	-	1
SY434	0.203	-	-	-	1
SR-CRSP-11	-	-	-	-	0
TGLA53	-	0.203	0.268	-	2
BM1258	-	-	-	-	0
SR-CRSP-9	-	-	-	-	0
ILSTS30	-	-	-	-	0
ETH225	-	-	-	-	0
SR-CRSP-6	0.213	0.276	-	-	2
BOBT24	-	-	-	-	0
NRAMP1	-	-	-	-	0
**Total**	**3**	**2**	**1**	**0**	**6**

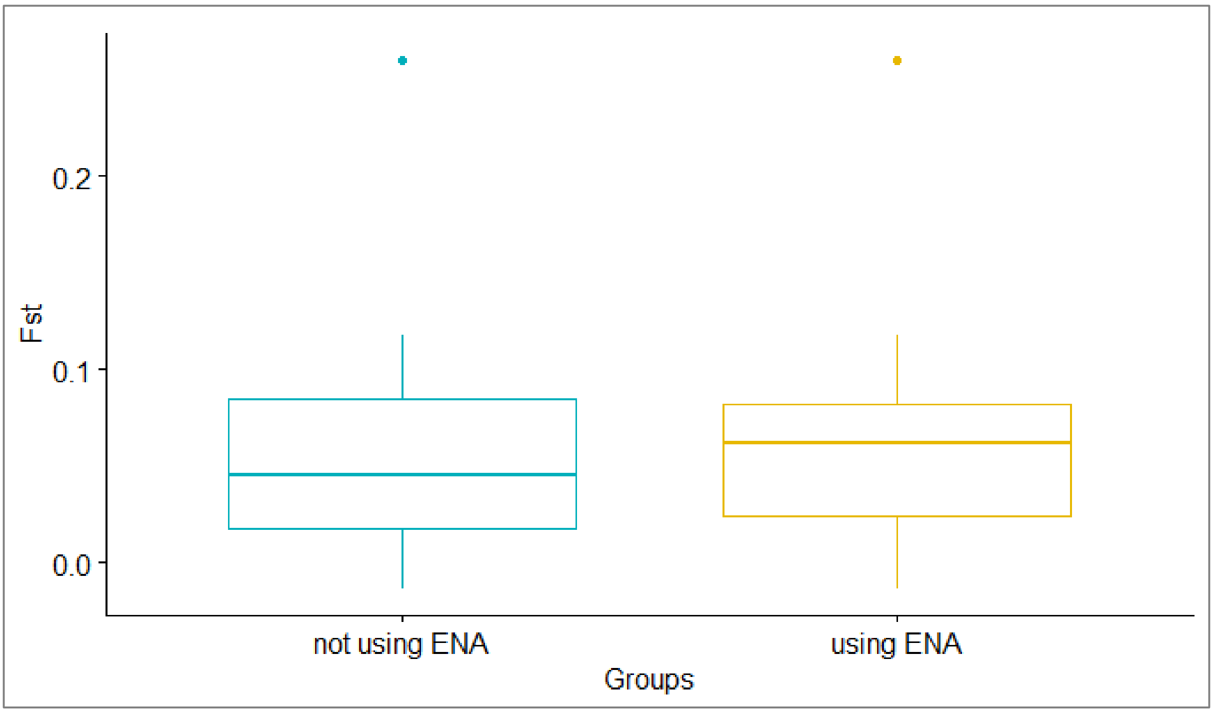

Figure S.1. The comparison of two groups of F_{ST} values (original pairwise F_{ST} values and ENA-corrected pairwise F_{ST} values) using the Wilcoxon Two Sample test.

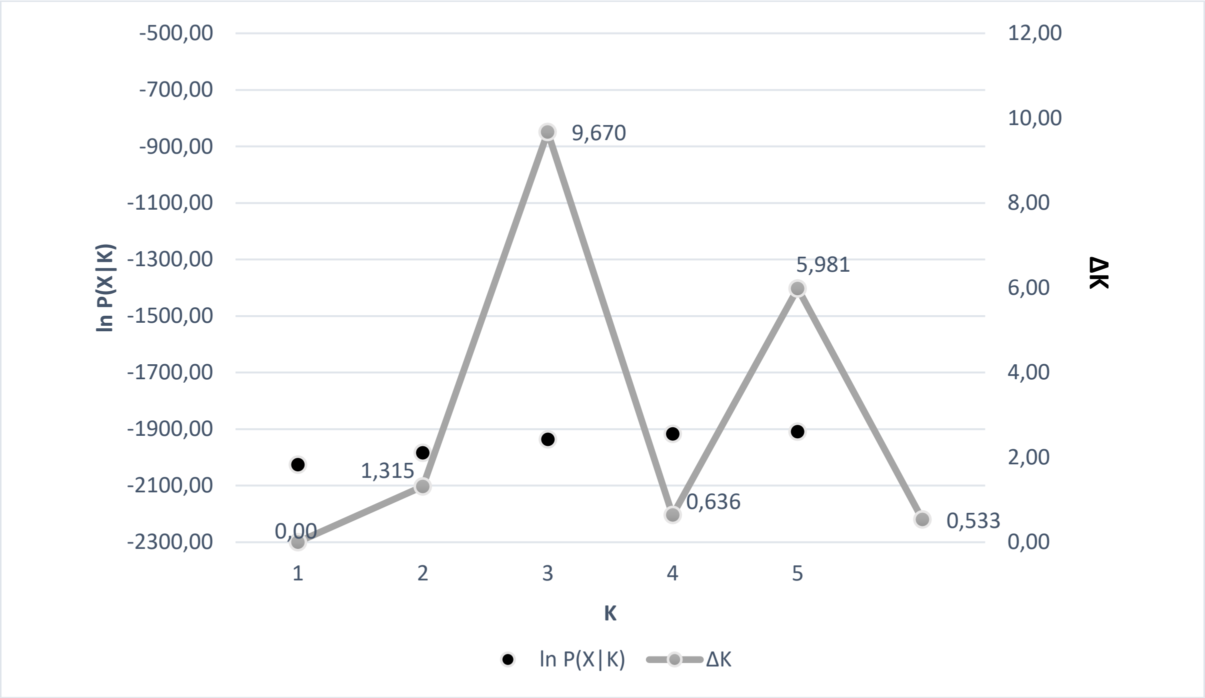

Figure S.2. Evanno method (Evanno et al. 2005) for selecting the representative number of clusters (*K*).

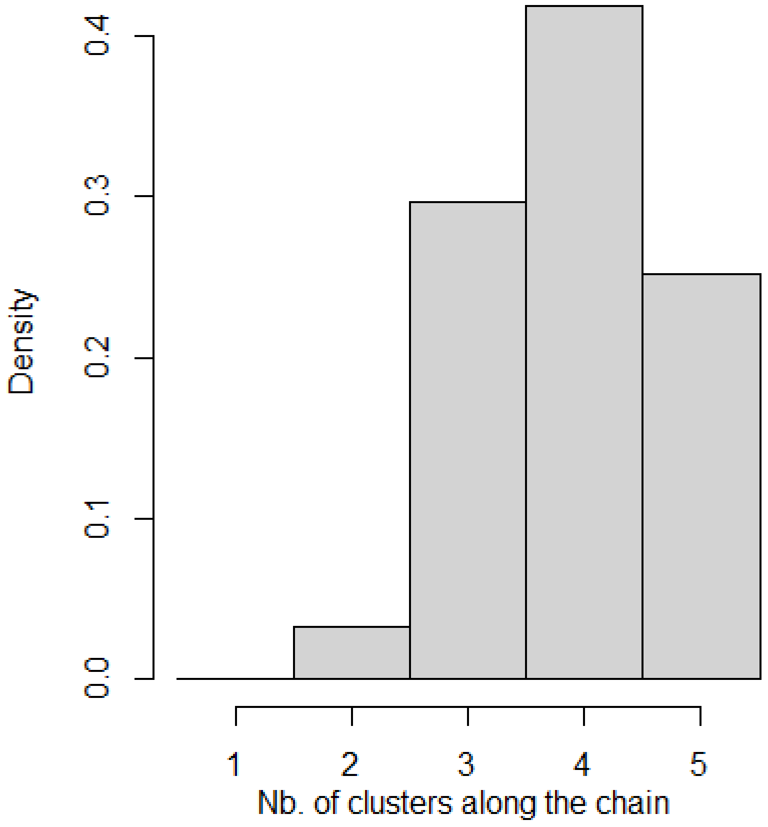

Figure S.3. Number of clusters along the MCMC chain for spatial analysis performed with the GENELAND software.
